# The melody of language learning at intermediate and upper levels: an emphasis on free discussion panels as an indispensable part of language classes and the effects on willingness to communicate, growth mindfulness, and autonomy

**DOI:** 10.1186/s40359-024-01645-5

**Published:** 2024-03-18

**Authors:** Jiaxue CAO, Xiaoshuang Liu

**Affiliations:** 1 Department of English Language, College of Foreign Languages, North China University of Science and Technology, Tangshan, Hebei Province 063210 PR China; 2 Tangshan Normal University, Tangshan, Hebei Province 063000 China

**Keywords:** Free discussion panels, Willingness to communicate, Growth mindfulness, Autonomy

## Abstract

This qualitative study investigated the impact of discussion panels on language education, focusing on willingness to communicate (WTC), growth mindfulness, and autonomy among Chinese learners at intermediate and upper-intermediate proficiency levels. The study, conducted in Hebei, China, involved 27 learners, with 14 in the experimental group exposed to discussion panels and 13 in the control group receiving traditional teacher-fronted lessons. The research design employed semi-structured interviews, observations, and document analysis for data collection, analyzed manually through thematic analysis. Results revealed that learners in the experimental group exhibited heightened WTC, increased growth mindfulness, and greater autonomy compared to the control group. The discussion panels facilitated authentic language use, collaborative discourse, and turn-taking, aligning with communication theory, sociocultural theory (SCT), and learner-centered pedagogy. Findings also resonated with the significance of WTC in language learning, supported by established theories. Additionally, the study contributes to the growing literature on the intersection of mindfulness, autonomy, and language education. Implications for language teachers, policy-makers, syllabus-designers, and materials developers are discussed, emphasizing the potential benefits of integrating discussion panels. The study concludes with insights into limitations, suggestions for further research, and a call for pedagogical innovation to enhance language learning experiences.

## Introduction

In an era marked by unprecedented global connectivity, the acquisition of a second language (L2) stands as a fundamental skill with far-reaching implications for personal, academic, and professional development. As language learners progress from intermediate to upper levels, the significance of fostering a nuanced and expressive linguistic competence becomes increasingly apparent [1]. Beyond the conventional realms of structured lessons and prescribed exercises, the cultivation of language proficiency requires an immersive and dynamic approach that transcends traditional pedagogical boundaries [2].

A panel discussion is a form of discourse where members of a specific group engage in conversations on a given topic, presenting diverse perspectives before an audience [3]. defines it as a platform for criticizing issues, problem-solving through argumentation, and brainstorming ideas. This researcher further specifies that participants focus on discussing questions relevant to them, fostering communication skills, consciousness, and activity [3]. Panel discussions, in this process, cultivate turn-taking conversational democracy, teaching students effective communication [4]. This method enhances students’ awareness of their social surroundings and the audience, contributing to the development of speaking abilities [4–5]. Employing a student-centered approach, teachers act as moderators and facilitators, guiding the class toward the goal of enhancing speaking skills [4]. This approach also fosters learner autonomy through peer collaboration, utilizing peer review to encourage active learning and focus on individual perspectives [6]. Consequently, learners develop a propensity to concentrate on their unique viewpoints.

The conceptualization of WTC initially pertained to L1 communication, defined by [7] as the intention to initiate communication when the choice is available. Subsequently, WTC was extended to L2 communication, adopting a situational construct due to the diverse range of potential L2 competencies and communication opportunities [8]. provided a definition of L2 WTC as ‘a readiness to enter into discourse at a particular time with a specific person or persons, using L2.’ They observed that L2 WTC varies with different interlocutors over time and in diverse situations. The dynamic nature of WTC is a result of the combined influence of linguistic, psychological, and contextual factors outlined in [8] WTC model, validated by studies in various contexts [9–10]. Long-term influences on WTC encompass personality traits, intergroup climate, communicative competence, and intergroup attitudes. Immediate influences involve state communicative self-confidence and the desire to communicate with a specific person.

As a comprehensive competence in Second Language Acquisition (SLA), mindfulness, characterized as an individual’s ability to focus attention on current external and internal events, experiences, and conditions in the present moment [11], assumes a pivotal role. To elaborate, the awareness cultivated through mindfulness practice extends to emotional skills, including emotional intelligence—an encompassing understanding of one’s own emotions and the capacity to recognize and empathize with others’ feelings. Mindfulness is also intricately linked to individuals’ perceptions of their environment, particularly the resources available, influencing academic competencies. [12–13] have substantiated that engaging in mindfulness enhances intellectual capabilities and encourages a receptivity to diverse modes of thinking. Moreover, as mindfulness facilitates emotional regulation, self-awareness, and a forward-looking orientation, its association with SLA is plausible [14–16].

At the heart of effective language learning lies the concept of autonomy, a dynamic force that empowers learners to take ownership of their educational journey [17]. Autonomy in language acquisition transcends the mere acquisition of vocabulary and grammar rules; it embodies a profound shift towards self-directed learning and individual agency [17]. In the context of language education, learners’ autonomy signifies the ability to make informed decisions, set personalized goals, and engage in self-regulated strategies that extend beyond the confines of the classroom [18]. As learners progress to intermediate and upper levels, the cultivation of autonomy becomes increasingly pivotal, propelling them toward a level of linguistic mastery that extends beyond rote memorization [18].

Free discussion panels were chosen as the intervention for several reasons. Firstly, they provide a dynamic and interactive environment that fosters active participation and engagement among language learners. By encouraging open dialogues and the exchange of diverse perspectives, free discussion panels promote authentic communication and meaningful interaction, which are essential for language acquisition. Additionally, free discussion panels offer opportunities for learners to practice and improve their listening and speaking skills in a supportive and collaborative setting. This intervention aligns with the principles of learner-centered pedagogy, as it empowers learners to take ownership of their learning process and enhances their autonomy. Moreover, free discussion panels allow for the exploration of complex topics and the negotiation of meaning, facilitating deeper cognitive processing and understanding. Overall, the choice of free discussion panels as the intervention reflects a holistic approach to language education, focusing on both linguistic proficiency and socio-cultural competence.

Despite the recognized significance of language acquisition at intermediate and upper levels, contemporary language education often encounters challenges in fostering comprehensive and expressive linguistic competence among learners. While conventional methods prioritize structured lessons and prescribed exercises, the need for an immersive and dynamic approach becomes increasingly apparent. Furthermore, the specific impact of incorporating free discussion panels, a form of discourse characterized by diverse perspectives and interactive engagement, in enhancing language proficiency and addressing cognitive and affective dimensions remains an underexplored area in the existing literature. This study seeks to address this gap by investigating the effects of free discussion panels on learners’ WTC, growth mindfulness, and autonomy within the context of intermediate and upper-level language classes. Through a meticulous examination of the intricate relationship between open dialogue and language acquisition, this research aims to contribute valuable insights to pedagogical practices and enrich the ongoing discourse on effective language learning strategies.

The primary objective of this study is to investigate the impact of integrating free discussion panels into intermediate and upper-level language classes. Specifically, the research aims to assess how the incorporation of these panels influences learners’ WTC, growth mindfulness, and autonomy. By exploring the nuanced dynamics between open dialogue and language acquisition, the study seeks to provide insights into effective pedagogical practices that foster comprehensive and expressive linguistic competence. Additionally, the research aims to contribute to the existing literature by offering a detailed examination of the transformative potential of free discussion panels in advancing language proficiency and addressing cognitive and affective dimensions among language learners at advanced stages. Through the implementation of qualitative methods, the study strives to illuminate the multifaceted effects of free discussion panels and provide practical implications for language educators and curriculum developers. Thus, the following research questions are addressed:

1. How does free discussion panels affect learners’ willingness to communicate?
2. How does free discussion panels influence learners’ mindfulness?
3. How does free discussion panels influence learners’ autonomy?

The significance of this study is paramount in light of the evolving landscape of language education and the essential role language acquisition plays in personal, academic, and professional development. While the importance of fostering linguistic competence is widely acknowledged, there exists a critical gap in understanding the specific impact of integrating free discussion panels into intermediate and upper-level language classes. The conventional emphasis on structured lessons and prescribed exercises may not fully capture the dynamic and immersive nature required for comprehensive language proficiency.

This study seeks to address this gap by delving into the intricate relationship between free discussion panels and language acquisition. By exploring the effects on learners’ WTC, growth mindfulness, and autonomy, the research aims to offer valuable insights into effective pedagogical practices that extend beyond traditional methodologies. The investigation into the transformative potential of free discussion panels holds promise for enhancing the communicative competence of language learners, promoting self-directed learning, and fostering nuanced and expressive linguistic mastery.

Furthermore, the study’s focus on the cognitive and affective dimensions of language learning at advanced stages contributes significantly to the ongoing discourse on effective language learning strategies. By delving into the intricate interplay between pedagogical approaches and learners’ cognitive and affective processes, the research questions address not only the immediate effects on communication willingness, mindfulness, and autonomy but also aim to provide a nuanced understanding of the multifaceted impact of free discussion panels. Consequently, the findings of this study hold considerable potential to inform language educators, curriculum developers, and researchers, offering practical implications for optimizing language education strategies and advancing the field of SLA. Through a meticulous examination of the interplay between open dialogue and language acquisition, this study aspires to contribute significantly to the enhancement of language learning practices in intermediate and upper-level language classes. The implications could extend beyond the classroom, potentially influencing educational policies and initiatives aimed at promoting learner-centered approaches and fostering socio-cultural competence in language learners. Policymakers could draw upon these findings to shape comprehensive language learning policies that prioritize communicative competence, learner autonomy, and socio-cultural awareness. Additionally, the research underscores the importance of ongoing professional development for language educators, equipping them with the skills and knowledge necessary to implement innovative pedagogical methods effectively. As the landscape of language education continues to evolve, this study serves as a valuable resource for practitioners and policymakers alike, guiding efforts to create dynamic and effective language learning environments that meet the diverse needs of learners.

## Literature review

### Theoretical background

#### Discussion panels

The integration of panel discussions into language education finds its theoretical underpinnings in communication theory, SCT, and learner-centered pedagogy. Drawing on Bakhtin’s concept of dialogism [19], which emphasizes the dialogic nature of language, panel discussions provide learners with a platform for authentic conversations. This form of discourse promotes language development through active participation, negotiation of meaning, and exposure to diverse linguistic expressions. Additionally, Vygotsky’s SCT [20], highlighting the social and cultural nature of learning, aligns with the practice of panel discussions as a means of fostering collaborative discourse. Within this SCT approach, learners engage in shared construction of knowledge through group interaction, reflecting the idea that language learning is deeply connected to meaningful social exchanges.

The integration of panel discussions also resonates with learner-centered pedagogy, advocating a shift from teacher-centered approaches to empower learners in their educational journey. Grounded in the principles of educational theorists such as Dewey [21] and Knowles [22], learner-centered methods emphasize experiential and participatory learning. In the context of panel discussions, teachers serve as facilitators, promoting student engagement, critical thinking, and the practical application of language skills in authentic, real-world scenarios.

Furthermore, the concept of turn-taking, rooted in conversation analysis [23], is integral to panel discussions. This practice promotes conversational democracy, ensuring equal opportunities for participants to express their thoughts and engage in dialogue. The emphasis on turn-taking contributes to the development of effective communication skills as learners navigate conversational dynamics, actively listen, and respond thoughtfully—a key aspect of language proficiency.

Finally, the integration of panel discussions fosters learner autonomy, as conceptualized by [24]. Autonomy in language learning involves active participation in decision-making processes, goal-setting, and self-regulated learning. Panel discussions provide opportunities for peer collaboration, peer review, and individual perspectives, empowering learners to take ownership of their language learning journey.

### Willingness to communicate

The concept of WTC originated in 1976 with Burgoon [25], who initially termed it “unwillingness to communicate.” Subsequently, [26] introduced this notion to the realm of second or foreign language teaching and learning. In the context of a second or foreign language, WTC entails actively seizing opportunities to engage in communication using the target language. Recognizing the significance of the desire to communicate is rooted in the fundamental objective of language teaching, which is to equip learners with the skills to articulate their intended meanings and concepts in the target language. Unwillingness to communicate poses a hindrance to the successful acquisition of a foreign language.

Furthermore, established linguistic theories like the Interaction Hypothesis, Socio-cultural Hypothesis, and Output Hypothesis underscore the pivotal role of interaction and communication in second language learning [27]. The motivation to communicate is integral to communicative teaching methods and group language learning, where collaborative efforts among learners and the use of a second or foreign language for interpersonal communication are paramount. Initially applied to first language learning by [26, 28] later observed that the tendency to communicate in a second or foreign language is not a fixed trait and can vary under different influences. Subsequently, an exploratory model, as presented by [29–30], highlights two categories of variables: transient variables, encompassing environmental factors such as classmates, teachers, and classroom atmosphere, and persistent variables, including anxiety and motivation.

### Growth mindfulness

The Pali terms sati and sampajañña, signifying the presence of mind and attentiveness to the current moment, serve as the origin for the term mindfulness [31]. While defining mindfulness can be challenging, earlier research has commonly adopted by [32]’s definition, characterizing it as the intentional act of paying attention in the present moment without judgment [33]. provide a more comprehensive definition, encompassing both mindful awareness and mindful practice, emphasizing mindfulness as both a process and an outcome. The former involves a structured program with key components such as intention, attention, and attitude, aiming at cultivating skills like sustained focus, acceptance, discernment, and compassion. The latter taps into an inherent human capacity for awareness, allowing objective recognition of life experiences contributing to well-being or distress, both personally and for others. Mindfulness enables a more flexible creation of autobiographical meaning, enhancing individuals’ ability to positively reflect on negative experiences and savor positive ones [34–35]. Numerous studies [36–38] indicate that mindfulness practice promotes well-being, strengthens interpersonal connections, alleviates stress, and serves as a preventive measure against burnout.

### Autonomy

Autonomy, as outlined in the Self-Determination Theory (SDT) [39–41], stands as one of the fundamental psychological needs with intrinsic motivational implications for personal development and well-being (ibid.). Within this framework, autonomy is defined as the behavior aligned with one’s personal beliefs, genuine interests, and values. The degree of autonomy in behavior regulation plays a critical role in performance, persistence, and overall well-being, serving as a central factor in motivating individuals [39–41].

Numerous studies, particularly within the realm of L2 learning, have affirmed the significant connection between autonomy and motivation (e.g., [42–45]), especially in online learning environments [46]. Autonomy emerges as a more influential predictor of proficiency compared to language anxiety and motivation in this particular context [47]. The digital learning landscape is acknowledged both as a necessity [48] and a potential enhancer of learners’ autonomy. Its affordances range from facilitating access to resources “anytime, anywhere” to heightening students’ awareness of the learning process [49] and fostering positive attitudes toward autonomous learning [50]. However, there is a noted caution regarding the potential risk of technology instilling a false sense of development in students [48].

### Empirical background

Previous investigations into the WTC have revealed that the disposition, assistance, and instructional approach of educators can impact the WTC of learners. In a qualitative investigation conducted by [51], a concentrated essay technique was employed to scrutinize the ways in which teachers can shape students’ inclination to engage in verbal communication during class. Participants in the study were requested to recount instances where teachers had an impact on their WTC in English. A total of 97 submissions were collected detailing situations where students were most inclined to communicate and 84 submissions for instances when students were least inclined to communicate. The results suggested that teachers’ delay in response, correction of errors, choice of discussion topics, and provision of support play a role in influencing the WTC of learners.

 [52] utilized a mixed methodology to examine L2 WTC as a dynamic factor within synchronous group discussion activities. Data were gathered from a cohort of Farsi-speaking ESL students participating in six online discussion sessions. Every 5 min throughout the discussions, participants assessed their WTC levels and subsequently provided rationales for shifts in WTC through written stimulated recall procedures. The findings revealed substantial fluctuations in participants’ WTC levels during the online discussion tasks, supporting the notion of online WTC as a constantly changing dynamic element. Furthermore, the results illustrated that alterations in WTC are instigated by intricate interactions involving various internal and external influences.

Past studies have extensively delved into elucidating enduring patterns and connections among variables at either the trait level or specific to situations [53]. distilled insights from the realms of language anxiety and language learning motivation to construct the argument that the act of initiating communication at a specific juncture can be construed as a deliberate, freely chosen process. The outcome was a level of WTC that has the potential to undergo rapid fluctuations in response to evolving circumstances. This discussion references prior research, employing both qualitative and quantitative approaches, to illustrate the intricacy inherent in the processes shaping WTC. The contention posited the necessity of adapting methodologies to focus on the dynamic process of deciding to engage or avoid L2 communication when presented with the opportunity.

 [54] investigated student interactions during mindful tasks in an English reading class, examining the potential link between mindfulness and effective interaction. Employing a case study approach, eight English language and literature BA students at the University of Mazandaran participated, divided into non-mindful and less mindful groups based on Mindful Attention Awareness Scale (MAAS) scores. Three sessions of critical reading practice were conducted, with the less mindful group engaging in mindfulness activities before tasks. Video recordings of all sessions were transcribed and analyzed by the researcher and a second rater. Results revealed minor differences in interactions between the non-mindful and less-mindful groups, with the less-mindful group exhibiting more interactions during critical reading tasks.

Social mindfulness involves respecting and safeguarding others’ choices in interpersonal communication. The object-choosing task, a conventional method for assessing social mindfulness, evaluates an individual’s preference for a nonunique object, allowing others more options, or a unique object, limiting choices for others. No previous research has explored how perceptions of individuals with varying levels of social mindfulness impact cooperation. To address this, two experiments were conducted by [55]. In both experiments, the social mindfulness of a confederate participant (Player A) was manipulated by varying the frequency of choosing unique and nonunique objects. Subsequently, participants engaged with Player A in either the public goods game (Experiment 1) or the centipede game (Experiment 2). The consistent findings indicated that participants interacting with someone perceived as socially mindful, in comparison to those interacting with a socially unmindful person, contributed more resources in the public goods game and chose to pass on more rounds in the centipede game.

The effectiveness of recently introduced language learning methods can be optimized through meticulous and regular monitoring within specific application contexts. To achieve this, educational programs should be scrutinized, and key elements of each learning context identified. Based on responses from 638 students, [56] developed and validated a scale to assess participants’ perceptions of how classroom interaction, learner needs, learner autonomy, pedagogical scaffolding, and learner identity could influence learning activities. Utilizing AMOS 22, survey results were imported to test and validate a hypothetical model of these variables. The validated model confirmed the interconnected relationships among the study variables, highlighting the significant role of interaction in regulating, predicting, shaping, and explaining behaviors of other variables. These findings contribute to understanding the sociocultural aspects of classroom interaction, learner needs, learner autonomy, pedagogical scaffolding, and learner identity in TEFL programs, promoting positive developments and widely accepted practices for improvement.

The prevailing notion suggests that residing in a country where the target language is spoken can lead to rapid improvement in L2 proficiency due to constant exposure in an immersion setting. Nevertheless, scholars contend that the effectiveness of this learning environment depends on individual students. Some may face limitations in accessing native speakers based on their circumstances, while others may feel uneasy interacting in the target language, possibly hindering the development of autonomy [57]. scrutinized learners facing such challenges when provided opportunities to converse with native speakers. Specifically, it investigated how regular interaction influenced ESL learners’ perspectives on language learning, as evident in their journal entries and interviews. The findings highlighted participants’ development of communicative strategies and positive shifts in perceptions toward language learning following these interactions.

The studies cited offer diverse perspectives on factors influencing WTC in L2 learners. While [51] underscores the role of teachers in shaping students’ inclination to engage in verbal communication through various classroom practices, [52] delves into the dynamic nature of WTC during online discussion tasks, highlighting the intricate interplay of internal and external influences [53]. contributes to this discourse by emphasizing the dynamic process of deciding to engage or avoid L2 communication, drawing from insights on language anxiety and motivation. Moreover, [54] explores the potential link between mindfulness and effective interaction, suggesting mindfulness activities may enhance engagement during language tasks. Additionally, [55] investigates social mindfulness and its impact on cooperation, offering insights into the socio-interpersonal aspects of communication. Lastly, [56] provides a comprehensive framework for assessing various factors influencing language learning activities, underscoring the significance of classroom interaction and learner autonomy. Conversely, [57] examines challenges faced by ESL learners in accessing native speakers, shedding light on the nuanced relationship between language immersion and learner autonomy. These studies collectively contribute to a holistic understanding of the multifaceted nature of WTC and its implications for language education practice and policy-making.

Despite the acknowledged significance of language acquisition at intermediate and upper levels, contemporary language education encounters challenges in nurturing comprehensive and expressive linguistic competence among learners. Conventional methods, characterized by structured lessons and prescribed exercises, may fall short of providing the dynamic and immersive approach needed for language proficiency. Additionally, the specific impact of integrating free discussion panels into language classes, characterized by diverse perspectives and interactive engagement, remains an underexplored area in the existing literature. This study aims to address this gap by investigating the effects of free discussion panels on learners’ WTC, growth mindfulness, and autonomy within the context of intermediate and upper-level language classes. Through a meticulous examination of the intricate relationship between open dialogue and language acquisition, this research seeks to contribute valuable insights to pedagogical practices and enrich the ongoing discourse on effective language learning strategies.

## Method

### Design

This qualitative study adopts a phenomenological approach to explore the experiences of language learners at intermediate and upper levels participating in free discussion panels. Purposive sampling will be used to select participants actively engaged in these panels, and data will be collected through in-depth interviews, participant observations, and document analysis. Thematic analysis will be applied to identify recurring patterns and themes. The study aims to uncover the subjective meanings attached to free discussion panels, providing insights for language educators and policymakers.

### Setting and participants

The study was set in a university in Hebei, China, focusing on learners at intermediate and upper-intermediate levels of language proficiency. Purposive sampling, a method chosen to ensure a diverse representation of language learners, was employed to select participants, ensuring a diverse representation of language learners. A total of 27 participants, aged between 18 and 23 years old, were involved, all sharing Chinese as their L1. Among them, 14 learners were designated for the experimental group (EG), while 13 were assigned to the control group (CG). Gender distribution included 4 male learners in the EG and 6 in the CG, with the remaining participants being female. Notably, none of the participants have ever visited an English-speaking country, setting a commonality in their language learning experiences and exposure. The selection criteria for participants were carefully designed to maximize the study’s internal validity and enhance the generalizability of its findings to similar language learning contexts. It is essential to pinpoint that the inclusion of a CG was essential to establish a baseline for comparison, allowing researchers to assess the specific effects of the intervention (free discussion panels) by contrasting it with traditional instructional methods. This design helps control for extraneous variables and increases the internal validity of the study, enabling researchers to draw more reliable conclusions about the effectiveness of the intervention on language learners’ outcomes.

Purposive sampling was utilized in this qualitative study to ensure the selection of participants who could provide rich and diverse insights into the research topic. However, the rationale behind the specific sample size of 27 participants and the allocation of 14 participants to the experimental group and 13 to the control group requires further elucidation. The determination of the sample size was influenced by factors such as the resources available for data collection and analysis, the anticipated depth of insights needed to address the research questions adequately, and the desire to achieve data saturation, where no new information emerges from additional participants. Additionally, the allocation of participants to the experimental and control groups was based on factors such as maintaining balance between the groups in terms of language proficiency levels, ensuring adequate representation of participants in each group, or logistical considerations.

### Instruments

The instruments employed in this qualitative study included semi-structured interviews [52, 56], participant observations, and document analysis. Semi-structured interviews served as the primary method of data collection, allowing for an in-depth exploration of participants’ experiences with free discussion panels. The interview guide was designed to capture insights into WTC, growth mindfulness, and autonomy, encouraging participants to reflect on their perceptions, challenges, and developments. Participant observations complemented the interviews, providing firsthand insights into the dynamics of free discussion panels, including non-verbal cues, interactions, and group dynamics. Lastly, document analysis involved the examination of reflective journals or artifacts produced by participants during or after the panel discussions, offering additional context and depth to their experiences. This multi-faceted approach aimed to provide a holistic understanding of the impact of free discussion panels on language learners’ subjective experiences.

### Data collection procedures

Data for this study were meticulously collected through a multifaceted approach, incorporating semi-structured interviews, participant observations, and document analysis. In the EG, participants engaged in dynamic free discussion panels meticulously designed to foster the development of listening and speaking skills. These panels provided a platform for participants to actively participate in open dialogues, enabling the sharing of diverse perspectives on predetermined topics. Semi-structured interviews were then conducted individually with each EG participant, delving into their nuanced experiences, perceptions, and challenges concerning WTC, growth mindfulness, and autonomy.

In contrast, participants in the CG received traditional teacher-fronted lessons characterized by structured instructional sessions following conventional pedagogical approaches. The CG participants also underwent individual semi-structured interviews, mirroring the methodology applied to the EG, thereby facilitating a robust comparative analysis of their educational experiences.

Furthermore, comprehensive participant observations were conducted during both the free discussion panels (EG) and traditional lessons (CG) to capture the intricacies of real-time interactions, subtle non-verbal cues, and dynamic group dynamics. These observations provided invaluable contextual insights into the learning environments of both groups.

Additionally, participants were encouraged to maintain reflective journals or produce artifacts documenting their experiences throughout the study duration. These reflective materials served as supplementary sources of data, offering deeper insights into participants’ cognitive and affective responses to the instructional methods employed.

To ensure the integrity and accuracy of data collection, semi-structured interviews were audio-recorded with participants’ explicit consent. Detailed field notes were diligently taken during participant observations to capture observations, thoughts, and reflections in real-time. Subsequently, the collected data were meticulously transcribed and meticulously organized for thematic analysis. This rigorous analytical approach facilitated a comprehensive exploration of the impact of different instructional methods on language learners’ WTC, growth mindfulness, and autonomy, thereby enriching the study’s findings and contributing to a nuanced understanding of language education practices.

Triangulation and integration of data were vital components of ensuring methodological rigor in this study. Multiple data sources, including semi-structured interviews, participant observations, and document analysis, were triangulated and integrated to provide a comprehensive understanding of the research phenomenon. For instance, findings from semi-structured interviews were compared and contrasted with observations made during participant observations. This comparison allowed for the validation of participants’ self-reported experiences with actual behaviors observed during free discussion panels or traditional lessons. Similarly, document analysis, which involved examining reflective journals or artifacts produced by participants, provided additional context and depth to the data collected through interviews and observations. By synthesizing information from these diverse sources, researchers were able to identify patterns, themes, and discrepancies, thereby enriching the overall analysis and enhancing the trustworthiness of the findings. Additionally, thematic analysis served as a central mechanism for integrating data, as recurring themes identified across different sources were synthesized to construct a coherent narrative that encapsulated the multifaceted experiences of language learners participating in discussion panels. This iterative process of comparison, contrast, and synthesis ensured methodological rigor by triangulating findings from varied perspectives and sources, ultimately strengthening the credibility of the study’s conclusions.

### Data analysis procedures

The data analysis procedures for this study involved a thorough examination of the collected data through semi-structured interviews, participant observations, and document analysis. The semi-structured interviews were transcribed verbatim from the audio recordings, ensuring accuracy in capturing participants’ responses. The transcriptions were then manually coded, and codes were grouped into emerging themes related to WTC, growth mindfulness, and autonomy.

Participant observations were documented through detailed field notes, capturing observations of participants’ behavior, interactions, and non-verbal cues during free discussion panels (EG) and traditional lessons (CG). These field notes were systematically reviewed, and patterns were identified through manual coding, contributing to the overall thematic analysis.

Document analysis involved the examination of reflective journals and artifacts produced by participants. These documents were manually coded to extract relevant themes, shedding light on participants’ individual perspectives and experiences.

Thematic analysis was conducted manually, involving the identification, coding, and categorization of recurring themes across all data sources. A coding framework was developed based on the identified themes, and data were organized accordingly. Patterns and relationships within and across themes were explored to derive meaningful interpretations of the impact of instructional methods on language learners’ WTC, growth mindfulness, and autonomy.

Throughout the analysis, careful attention was given to ensuring the trustworthiness and credibility of the findings, with the researchers engaging in ongoing discussions to refine the coding framework and interpretations. The manual analysis process allowed for a rich and detailed exploration of participants’ experiences and contributed to the depth of understanding of the study.

## Results

### The potential impact of free discussion panels on learners’ willingness to communicate

#### Semi-structured interviews

The results gleaned from the semi-structured interviews offer nuanced insights into the potential impact of free discussion panels on learners’ WTC. Participants from the EG, who engaged in discussion panels emphasizing listening and speaking skills, consistently expressed heightened motivation to communicate in the target language. They noted an increased sense of confidence in expressing their ideas, contributing to a more positive language-learning experience. The open and interactive nature of the discussion panels was identified as a key factor in fostering a supportive environment that encouraged participants to overcome communication apprehension.

Student A, who actively participated in free discussion panels, expressed a notable improvement in WTC. During the semi-structured interview, Student A conveyed a heightened sense of confidence in expressing thoughts and opinions in English. The interactive nature of the discussion panels contributed to overcoming initial communication apprehension, fostering a positive and encouraging atmosphere. Student A highlighted the significance of engaging in open dialogues as a catalyst for improved language use and interpersonal communication skills.

Student B, another participant in the EG, echoed similar sentiments during the interview. Having actively engaged in discussion panels emphasizing listening and speaking skills, Student B reported a positive shift in WTC. The open and collaborative environment of the panels provided a platform for sharing diverse perspectives, which, in turn, enhanced language confidence. Student B emphasized that the experience not only improved language skills but also contributed to a more enjoyable language-learning journey.

In contrast, participants from the CG, exposed to traditional teacher-fronted lessons, often articulated challenges related to WTC. They mentioned feeling less motivated to actively participate in class discussions and perceived a lack of opportunities for authentic language use. The teacher-centric approach in the CG was highlighted as a potential barrier to their engagement, hindering the development of a robust WTC in the target language.

Student C, who experienced traditional teacher-fronted lessons, expressed challenges related to WTC during the interview. The teacher-centric approach in the CG was identified as a potential barrier, limiting opportunities for active participation. Student C noted feeling less motivated to engage in class discussions and expressed a desire for more interactive language use. This student’s responses highlighted the impact of instructional methods on the development of willingness to communicate in the traditional setting.

Student D, another member of the CG, shared similar sentiments during the semi-structured interview. Reflecting on the traditional lessons, Student D articulated a sense of hesitancy in expressing ideas and opinions in the target language. The lack of interactive elements and authentic language use in the classroom setting emerged as factors influencing a lower willingness to communicate. Student D underscored the importance of fostering a more dynamic and participatory learning environment for improved language engagement.

Overall, the semi-structured interviews revealed a promising trend in the EG, suggesting that free discussion panels may positively influence learners’ WTC by creating an environment that promotes active participation, confidence-building, and meaningful interaction in the target language. These findings underscore the potential pedagogical benefits of incorporating discussion panels in language learning settings.

### Observation

The observation results shed light on the potential impact of free discussion panels on learners’ WTC, providing valuable insights into the dynamics of language engagement within the EG and the CG.

Participants in the EG exhibited increased participation and engagement during free discussion panels. The open nature of the discussions created an environment where learners felt encouraged to actively contribute, share their perspectives, and engage in spontaneous conversations. Observable behaviors, such as increased body language and eye contact, indicated a higher WTC among EG participants.

Noteworthy was the enhanced confidence displayed by EG participants in expressing ideas and opinions. The interactive format of the discussion panels allowed learners to practice and refine their language skills in a supportive setting. Increased verbal contributions and a willingness to initiate discussions suggested a positive impact on learners’ confidence levels when communicating in the target language.

Observations in the CG revealed comparatively limited participation and interaction during traditional teacher-fronted lessons. The teacher-centric approach tended to restrict opportunities for learners to actively engage in discussions. Instances of reduced body language and fewer spontaneous interactions indicated a potential impact on WTC within the CG.

In the CG, lower verbal engagement was evident during observations. Learners displayed hesitancy in expressing ideas, with a noticeable reliance on teacher prompting for responses. The structured nature of the lessons, while effective in delivering content, appeared to have an impact on the spontaneous verbal contributions and overall WTC among CG participants.

Overall, the observational results suggested that the interactive and collaborative nature of free discussion panels in the EG positively influenced learners’ WTC. In contrast, the more structured and teacher-centric approach in the CG presented challenges in fostering active language engagement among participants.

### Document analysis

The document analysis results provided additional perspectives on the potential impact of free discussion panels on learners’ WTC, offering insights from reflective journals and artifacts produced by participants in both the EG and the CG.

Participants in the EG consistently reflected positively on their WTC in the documents. Reflective journals highlighted instances where learners expressed a growing sense of confidence and willingness to actively communicate in the target language. The open and collaborative nature of the discussion panels emerged as a common theme, fostering an environment that encouraged learners to overcome communication apprehension and participate more freely.

The analysis revealed a notable improvement in verbal expression within the EG documents. Learners articulated instances where the discussion panels facilitated the development of their speaking skills, leading to increased comfort in expressing ideas. The dynamic and interactive nature of the panels played a pivotal role in enhancing participants’ WTC, as documented in their reflections.

Documents from the CG highlighted challenges related to WTC expression. Learners in this group expressed hesitancy in actively participating in class discussions and articulating ideas. The more traditional teacher-fronted lessons appeared to pose obstacles to their WTC, as documented in their reflections and observations of limited opportunities for authentic language use.

A common theme in CG documents was a desire for more interactive learning experiences. Participants expressed a longing for environments that allowed for increased verbal engagement, similar to the dynamic discussions reported by the EG. Documented reflections emphasized a preference for instructional methods that encourage active participation and foster a positive impact on WTC.

In summary, the document analysis results echoed the trends observed in both the interviews and observations. The EG documents highlighted positive shifts in learners’ willingness to communicate, attributing the improvement to the interactive nature of free discussion panels. In contrast, the CG documents revealed challenges and a desire for more interactive learning experiences to enhance WTC among participants.

### Identified themes

Several overarching themes emerged from the integrated analysis of semi-structured interviews, observations, and document analysis, collectively providing a comprehensive understanding of the potential impact of free discussion panels on learners’ WTC. The identified themes are as follows:

#### Increased confidence through free discussion panels

Across all data sources, there was a consistent theme of increased confidence among learners who participated in free discussion panels. Engaging in dynamic and interactive discussions positively influenced participants’ confidence levels, fostering a more positive outlook on expressing ideas and opinions in the target language.

#### Active participation in the EG

The EG consistently exhibited higher levels of active participation during free discussion panels. Observable behaviors and documented reflections indicated a willingness to actively engage in conversations, contributing to a supportive and collaborative learning environment.

#### Challenges in traditional teacher-fronted lessons

Participants in the CG, exposed to traditional teacher-fronted lessons, faced challenges in terms of willingness to communicate. The structured nature of these lessons and limited opportunities for authentic language use contributed to hesitancy and reduced verbal engagement among CG learners.

#### Desire for more interactive learning in the CG

Documented reflections from the CG revealed a collective desire for more interactive learning experiences. Participants expressed a preference for instructional methods that encourage active participation, similar to the dynamic discussions reported by their counterparts in the EG.

#### Positive impact on verbal expression

A prevalent theme in both document analysis and interviews was the positive impact of free discussion panels on learners’ verbal expression. Participants consistently reported improvements in their speaking skills, attributing these gains to the dynamic and collaborative nature of the discussion panels.

#### Role of teacher-centered approach in the CG

The teacher-centric approach in the CG emerged as a factor influencing the overall willingness to communicate. Observations and document analysis highlighted instances where a more traditional instructional approach appeared to limit opportunities for learners to express themselves freely.

#### Preference for collaborative learning environments

Learners across both groups expressed a preference for collaborative learning environments. Documented reflections and interviews underscored the importance of creating spaces that encourage open dialogue, peer collaboration, and a supportive atmosphere for language expression.

### Impact on overall language learning experience

A recurring theme across data sources was the impact of instructional methods on the overall language learning experience. Participants perceived free discussion panels as contributing positively to their language journey, making the learning process more enjoyable and meaningful.

These identified themes collectively contribute to a nuanced understanding of the multifaceted relationship between instructional methods, collaborative learning environments, and learners’ WTC in the context of language education.

### The potential impact of free discussion panels on learners’ growth mindfulness

#### Semi-structured interviews

In the semi-structured interviews conducted with participants from the EG, a consistent theme emerged regarding the heightened sense of self-awareness. Learners reported that engagement in free discussion panels facilitated an increased awareness of their thoughts, feelings, and reactions during language interactions. This introspective process was seen as a positive outcome contributing to the overall development of growth mindfulness. Additionally, participants expressed the development of reflective practices as a direct result of their involvement in free discussion panels. The open nature of the discussions encouraged learners to reflect on their own perspectives and consider alternative viewpoints, fostering a mindful approach to language use and communication. Another noteworthy finding was the participants’ shift in mindset towards challenges. Engaging in open discussions allowed learners to view linguistic challenges as valuable learning opportunities, aligning with the principles of growth mindfulness that emphasize a positive approach to challenges and continuous learning.

Conversely, interviews with participants from the CG revealed distinct patterns in their experiences. Limited development of self-awareness was reported, with learners expressing that the traditional teacher-fronted lessons did not provide as many opportunities for self-reflection and awareness of their own language learning process. Furthermore, participants in the CG tended to view linguistic challenges as obstacles rather than as opportunities for growth. The structured nature of the lessons and limited exposure to varied perspectives contributed to a more fixed mindset, hindering the development of a growth-oriented mindfulness approach. Additionally, CG participants reported less engagement in reflective practices compared to their counterparts in the EG. The absence of open discussions and collaborative exchanges limited opportunities for learners to reflect on their language use, resulting in a less reflective and mindful approach to the learning process.

Despite these differences, both groups acknowledged the potential impact of instructional methods on mindful language use. EG participants reported a positive influence on their language choices and expression, while CG participants expressed challenges in applying mindfulness principles to their language learning journey. Furthermore, participants from both groups perceived a connection between mindfulness and communication skills. EG learners associated their increased mindfulness with enhanced communication abilities, emphasizing the interplay between mindfulness practices and effective language expression. Overall, the semi-structured interviews provided nuanced insights into the varied experiences of growth mindfulness among learners in the context of free discussion panels and traditional teacher-fronted lessons.

### Observation

Observations of learners in the EG during free discussion panels revealed consistent patterns indicative of growth mindfulness. One prominent observation was the increased engagement in reflective practices among EG participants. As they actively participated in open discussions, learners demonstrated a heightened awareness of their language choices, actively reflecting on the nuances of their expressions and considering alternative perspectives. This reflective engagement contributed to an atmosphere where learners embraced challenges as learning opportunities. The open and dynamic nature of the discussion panels facilitated a positive shift in mindset, with participants demonstrating a willingness to approach linguistic challenges with curiosity and adaptability.

In contrast, observations of the CG during traditional teacher-fronted lessons presented a different picture. Limited engagement in reflective practices was noted, as the structured nature of the lessons provided fewer opportunities for learners to actively reflect on their language use. Challenges were often viewed as obstacles rather than opportunities for growth, reflecting a more fixed mindset. Observations further revealed a tendency among CG participants to exhibit less spontaneous and reflective language choices, with a more cautious approach to verbal expression.

Across both groups, the impact of instructional methods on mindful language use was evident. EG participants consistently demonstrated a mindful approach to communication during open discussions, actively considering their language choices. In contrast, CG participants exhibited challenges in applying mindfulness principles to their language learning journey within the confines of traditional lessons. Additionally, the connection between mindfulness and communication skills was perceptible in both groups, with EG learners associating increased mindfulness with enhanced communication abilities.

In summary, the observational findings align with the outcomes of semi-structured interviews, providing a holistic understanding of the potential impact of free discussion panels on learners’ growth mindfulness. The dynamic and interactive nature of the panels in the EG fostered an environment conducive to reflective practices and a growth-oriented mindset, while the structured lessons in the CG presented limitations in cultivating similar growth mindfulness among participants.

### Document analysis

In the document analysis, an examination of written reflections and self-assessments from learners in both the EG and CG provided valuable insights into the impact of free discussion panels on growth mindfulness.

Participants in the EG consistently showcased enhanced reflective writing in their documents. Describing moments of heightened self-awareness during free discussion panels, learners expressed how the open format encouraged them to reflect on language choices and consider alternative viewpoints. This aligns with the observed development of growth mindfulness as noted in interviews and observations. Additionally, EG documents reflected a positive shift in mindset towards challenges, demonstrating a newfound willingness to embrace linguistic difficulties as integral to the learning process. The document analysis reinforced the notion that the collaborative nature of discussion panels contributed significantly to a growth-oriented mindset among EG learners.

Documents from the CG exhibited limited reflection on language use, with a focus on content covered in traditional teacher-fronted lessons rather than reflective practices. This suggested a constrained opportunity for self-awareness within the traditional lesson structure. Challenges were consistently portrayed as obstacles rather than opportunities for growth in CG documents. Participants expressed frustration with linguistic difficulties but provided fewer instances of adaptive and positive responses to challenges compared to their counterparts in the EG. The document analysis highlighted potential limitations in the development of a growth-oriented mindset within traditional lesson structures.

Correlating with the outcomes of semi-structured interviews and observations, the document analysis findings revealed consistent patterns. EG documents echoed themes of increased self-awareness, positive mindset shifts, and reflective engagement observed and reported by participants. In contrast, limitations in self-awareness and a more fixed mindset were evident in CG documents, aligning with observations and interview results. Both sets of documents provided insights into the implications for mindful language use. EG participants explicitly connected heightened mindfulness with improved language expression, demonstrating a tangible link between discussion panel formats and mindful language choices. Conversely, CG participants exhibited challenges in applying mindfulness principles to their language learning journey, emphasizing the potential impact of instructional methods on growth mindfulness.

In conclusion, the document analysis reinforced the consistent patterns observed in interviews and observations, providing a comprehensive understanding of the impact of free discussion panels on learners’ growth mindfulness. The findings underscored the role of discussion panels in fostering reflective practices, promoting a positive mindset, and influencing mindful language use among language learners.

### Identified themes

#### Enhanced reflective practices

Across semi-structured interviews, observations, and document analysis, a consistent theme emerged regarding the enhanced reflective practices fostered by free discussion panels. Participants in the EG demonstrated an increased ability to reflect on their language choices, consider alternative viewpoints, and engage in introspective analyses of their communication during open discussions.

#### Positive shift in mindset

Participants in the EG consistently exhibited a positive shift in mindset towards challenges within the free discussion panel format. This theme, identified through interviews, observations, and document analysis, highlighted how learners in the EG began viewing linguistic challenges as opportunities for growth, demonstrating a more adaptive and resilient mindset.

#### Heightened self-awareness

The theme of heightened self-awareness emerged prominently across all three data collection methods. EG participants, engaged in free discussion panels, demonstrated an increased awareness of their language choices, nuances in expression, and the impact of their communicative decisions. This self-awareness contributed to a more mindful approach to language use.

#### Collaborative learning environment

The collaborative nature of free discussion panels emerged as a central theme in fostering growth mindfulness. Learners in the EG engaged in collaborative discourse, actively negotiating meaning, and sharing diverse perspectives. This collaborative learning environment was consistently associated with the development of growth-oriented mindsets and increased mindfulness.

#### Challenges as learning opportunities

Both observations and document analysis highlighted a recurrent theme related to learners in the EG perceiving challenges as learning opportunities. The open and dynamic nature of discussion panels encouraged participants to approach linguistic difficulties with curiosity, adaptability, and a proactive orientation, contributing to the development of growth mindfulness.

#### Limited reflective practice in CG

In contrast, the CG demonstrated limited reflective practices within the traditional teacher-fronted lesson structure. This theme emerged across interviews, observations, and document analysis, indicating that the structured nature of traditional lessons presented fewer opportunities for learners to actively reflect on their language use.

#### Challenges views as obstacles in CG

Parallel to the positive shift observed in the EG, the CG exhibited a contrasting theme where challenges were often viewed as obstacles rather than opportunities for growth. This theme, identified through interviews, observations, and document analysis, suggested that learners in the CG faced challenges with less adaptability and resilience.

In summary, the identified themes collectively provide a comprehensive understanding of the potential of free discussion panels in fostering growth mindfulness among language learners. The collaborative and reflective aspects of the panel format contributed to a positive shift in mindset, increased self-awareness, and a transformative approach to challenges, highlighting the nuanced interplay between instructional methods and learners’ cognitive and affective dimensions.

### The potential impact of free discussion panels on Learners’ autonomy

#### Semi-structured Interviews

In exploring the perceived impact of free discussion panels on learners’ autonomy, participants from the EG consistently reported a heightened sense of decision-making agency. Engaging in open discussions empowered learners to actively make choices concerning the topics discussed, their level of participation, and the perspectives they contributed. The collaborative and open nature of the discussion panels fostered an environment conducive to increased autonomy, allowing participants to shape the direction of their language learning journey.

A notable theme that emerged from the interviews was the shift towards personalized goal-setting among learners in the EG. The exploratory nature of the discussion panels provided participants with the freedom to identify areas for improvement based on their unique language learning needs. This shift towards personalized goal-setting reflected a departure from more rigidly structured traditional lessons, providing learners with autonomy in determining their learning objectives.

Participants consistently expressed a sense of ownership over their language learning process as a result of engaging in free discussion panels. The interactive and dialogic nature of the panels allowed for active contributions to the direction and content of conversations. This ownership was indicative of a deeper engagement with language learning beyond the confines of the classroom, aligning with the concept of autonomy as an active and participatory endeavor.

Collaborative learning and peer influence emerged as integral components of autonomy within the EG. Participants highlighted the interactive nature of discussion panels, facilitating peer collaboration, idea-sharing, and mutual support. Autonomy, in this context, was perceived as a collective effort where learners actively contributed to and benefited from the collaborative learning environment, emphasizing the social dimension of autonomous language learning.

Learners in the EG reported a perceptible shift from passive to active learning experiences facilitated by free discussion panels. The autonomy cultivated through these panels encouraged participants to actively engage in discussions, express opinions, and take initiative in shaping the learning environment. This shift was contrasted with more passive roles often associated with traditional instructional methods, highlighting the transformative impact of discussion panels on learner engagement.

Autonomy emerged not only as a developmental aspect but also as a motivational factor for learners in the EG. The ability to actively participate in discussions, set personalized goals, and make informed decisions contributed to a sense of motivation and enthusiasm for language learning. These motivational aspects were consistently linked to the autonomy cultivated through engagement with free discussion panels, underscoring the interconnectedness of autonomy and learner motivation. In conclusion, the findings provide a nuanced understanding of how free discussion panels can positively impact learners’ autonomy, encompassing decision-making agency, personalized goal-setting, ownership of the learning process, collaborative learning, a shift towards active learning, and enhanced motivation.

#### Observation

Observations conducted during the integration of free discussion panels into language classes provided valuable insights into the potential impact on learners’ autonomy. In the EG, learners consistently demonstrated active participation and engaged in decision-making processes during free discussion panels. This manifested in their choice of topics, initiation of discussions, and collaborative decision-making within the group. The observed autonomy reflected learners’ ability to take ownership of their learning experience.

Additionally, observation revealed instances where learners in the EG employed self-directed learning strategies during and after free discussion panels. Participants were observed seeking additional resources, conducting independent research on discussed topics, and incorporating self-selected materials into their language learning process. This autonomy in seeking and utilizing resources showcased a proactive approach to learning.

Furthermore, learners in the EG demonstrated a higher degree of adaptability and flexibility in response to dynamic discussion scenarios. Observation indicated that participants navigated through diverse linguistic challenges with resilience, adjusting their communication strategies based on the evolving context. This adaptability showcased a form of autonomy linked to the ability to make real-time decisions in communicative situations.

The observation also highlighted instances where learners in the EG engaged in goal-setting discussions and reflective practices during and after panel sessions. Participants were observed setting personal language learning goals, discussing progress, and reflecting on areas for improvement. This proactive approach to goal-setting and reflection aligned with the autonomy cultivated through free discussion panels.

Moreover, the collaborative nature of autonomy emerged prominently during observations. Learners in the EG collaborated in setting group goals, negotiating language use, and collectively deciding the direction of discussions. This collaborative autonomy demonstrated the interplay between individual autonomy and the ability to engage collaboratively within a group setting.

In contrast, observations in the CG, where traditional teacher-fronted lessons were conducted, revealed a more limited expression of autonomy. Participants in the CG exhibited less initiative in topic selection, decision-making, and self-directed learning during structured lessons. The observed limited autonomy underscored the contrast between traditional instructional methods and the autonomy-rich environment of free discussion panels.

In conclusion, the observational findings suggested that free discussion panels facilitated a dynamic environment where learners exercised autonomy through active participation, self-directed learning strategies, adaptability, goal-setting, reflective practices, and collaborative decision-making. These observations contribute to a comprehensive understanding of how discussion panels can serve as a catalyst for fostering various dimensions of learner autonomy within language education.

### Document analysis

Document analysis yielded valuable insights into the impact of free discussion panels on learners’ autonomy. Examination of documents, including learners’ reflections, goal-setting exercises, and self-assessment reports, provided a nuanced understanding of the development of autonomy within the EG.

Participants in the EG consistently expressed a sense of ownership and control over their language learning journey through written reflections. These documents highlighted instances where learners actively made decisions about the topics they wanted to explore during discussion panels, reflecting a self-directed approach to learning. The language used in the reflections emphasized the learners’ agency, indicating a shift towards autonomy in decision-making processes.

Goal-setting exercises, a key component of document analysis, revealed a notable trend in the EG participants taking charge of their learning objectives. Learners articulated specific, personalized language learning goals, demonstrating a proactive stance in shaping their linguistic competencies. The goals set by participants often extended beyond the immediate classroom context, indicating a broader engagement with language learning beyond structured lessons.

Self-assessment reports provided additional evidence of the cultivation of autonomy within the EG. Learners consistently evaluated their own progress, identified areas for improvement, and proposed strategies for enhancement. These reports showcased a heightened self-awareness and a capacity for self-regulation, aligning with the attributes of learner autonomy.

Conversely, documents from the CG, primarily comprising traditional lesson plans, showed a more limited expression of learner autonomy. Reflections from the CG often focused on the content covered in class rather than personal insights or decisions. Goal-setting exercises were less individualized, with learners predominantly following predetermined objectives set by the instructor. The self-assessment reports in the CG reflected a more passive role, with learners relying on external evaluations rather than actively participating in self-reflection.

In summary, document analysis provided rich qualitative data illustrating the development of learner autonomy within the EG. The documents underscored learners’ active decision-making, personalized goal-setting, and self-regulation, contributing to a comprehensive understanding of the transformative potential of free discussion panels in fostering autonomy within language education.

### Identified themes

#### Active decision-making

Across various data sources, a recurring theme was the active engagement of learners in decision-making processes. Participants in the EG consistently demonstrated a proactive approach in choosing discussion topics, shaping the direction of conversations, and making decisions that influenced their learning experience. This theme emphasized the cultivation of autonomy through learners’ assertive involvement in the educational process.

#### Self-directed learning strategies

Another prominent theme was the adoption of self-directed learning strategies within the EG. Learners exhibited a tendency to independently seek additional resources, conduct research, and incorporate supplementary materials into their language learning journey. This theme highlighted autonomy manifested through learners’ initiatives in managing their own learning resources and strategies.

#### Goal-setting and reflection

The theme of goal-setting and reflection emerged consistently in the EG, indicating a strong connection to learner autonomy. Participants actively set personalized language learning goals, reflecting a sense of ownership over their educational objectives. The theme emphasized the role of reflective practices in fostering autonomy, as learners critically assessed their progress and formulated strategies for improvement.

#### Adaptability and flexibility

Observations and reflections indicated a theme of adaptability and flexibility within the EG. Learners demonstrated a capacity to navigate through linguistic challenges, adjusting their communication strategies based on the evolving context of discussion panels. This theme underscored autonomy in real-time decision-making and adaptability to varying communicative situations.

#### Collaborative autonomy

Collaborative autonomy emerged as a distinctive theme observed in the EG. Learners actively engaged in collaborative decision-making, negotiated language use within the group, and collectively shaped the direction of discussions. This collaborative aspect emphasized the interconnectedness of individual autonomy and the ability to collaborate effectively within a group setting.

#### Limited autonomy in CG

A contrasting theme emerged in the CG, where a more limited expression of learner autonomy was observed. Participants in the CG exhibited less initiative in decision-making, self-directed learning, and personalized goal-setting. This theme highlighted the distinction between traditional instructional methods and the autonomy-rich environment cultivated through free discussion panels in the EG.

These identified themes collectively provide a comprehensive understanding of the multifaceted nature of learner autonomy within the context of integrating free discussion panels into language education. The themes reflect the transformative potential of dynamic, learner-centered approaches in fostering autonomy and individual agency in the language learning process.

## Discussion

The findings of this qualitative study illuminate the transformative potential of integrating free discussion panels into language education, particularly concerning learners’ willingness to communicate, growth mindfulness, and autonomy. The observed effects provide valuable insights into the dynamic nature of language acquisition, shedding light on the nuanced interplay between pedagogical approaches and learners’ cognitive and affective dimensions.

The observed increase in learners’ willingness to communicate within the EG aligns with existing literature on the impact of learner-centered, interactive approaches in language education. The participatory nature of free discussion panels encourages learners to actively engage in discourse, contributing to the development of effective communication skills. The findings support the notion that creating a communicative and collaborative environment enhances learners’ confidence and motivation to initiate communication in the target language.

The incorporation of mindfulness principles within free discussion panels demonstrated a positive influence on learners’ growth mindfulness. Mindfulness practices, including attention to the present moment, emotional regulation, and self-awareness, were observed to contribute to a conducive learning environment. The findings resonate with previous research linking mindfulness to enhanced cognitive and emotional skills, which, in turn, can positively impact language learning experiences. The observed connection between mindfulness and language learning underscores the holistic nature of language acquisition, encompassing both cognitive and affective dimensions.

The central theme emerging from the study revolves around the cultivation of learner autonomy within the EG. The active decision-making, self-directed learning strategies, goal-setting, and adaptability observed among learners in the EG signify a profound shift toward autonomy. These findings align with the principles of learner-centered pedagogy, emphasizing the importance of empowering learners to take ownership of their educational journey. The observed limited expression of autonomy in the CG further underscores the contrast between traditional instructional methods and the autonomy-rich environment facilitated by free discussion panels.

The collaborative nature of autonomy within the EG highlights the interconnectedness of individual autonomy and the ability to collaborate effectively within a group setting. This collaborative autonomy is particularly noteworthy in the context of language learning, where communication is inherently social. The findings suggest that free discussion panels not only foster individual autonomy but also promote a shared construction of knowledge through group interaction, aligning with sociocultural theories of learning.

The collaborative environment fostered within the free discussion panels played a pivotal role in shaping the study’s findings by promoting authentic communication among participants. By encouraging open dialogue and active participation, the panels provided a platform for language learners to engage in meaningful exchanges, share diverse perspectives, and negotiate meaning collaboratively. This emphasis on authentic communication allowed participants to express themselves freely, experiment with language use, and explore different linguistic strategies in a supportive and non-judgmental setting. As a result, the data collected during these discussions offered rich insights into the participants’ language learning experiences, perceptions, and challenges related to willingness to communicate, growth mindfulness, and autonomy. The collaborative nature of the panels facilitated the emergence of nuanced themes and patterns, providing a deeper understanding of the impact of instructional methods on language learners’ cognitive and affective dimensions. Overall, the collaborative environment created within the free discussion panels contributed significantly to the study’s findings by fostering authentic communication and facilitating a rich exploration of language learners’ experiences.

During the data analysis process, several unexpected findings and divergent perspectives emerged, adding depth and complexity to the study’s insights. One unexpected finding was the varying degrees of engagement observed among participants within the EG during free discussion panels. While some learners exhibited high levels of enthusiasm and active participation, others appeared more reserved or hesitant to contribute to the dialogue. This divergence in engagement levels prompted further exploration into individual factors such as personality traits, prior language learning experiences, and comfort levels with speaking in a group setting. Additionally, divergent perspectives emerged regarding the perceived benefits of mindfulness practices within the context of language learning. While some participants expressed a profound appreciation for the mindfulness techniques incorporated into the discussion panels, citing improved focus, reduced anxiety, and heightened self-awareness, others remained skeptical or indifferent to its relevance. These contrasting viewpoints underscored the nuanced relationship between mindfulness and language learning outcomes, highlighting the importance of considering individual differences and preferences when implementing such strategies. Overall, these unexpected findings and divergent perspectives enriched the study’s findings by prompting deeper reflection and contextualization of the observed phenomena, thereby contributing to a more comprehensive understanding of the complex dynamics inherent in language education.

Comparing our study with the investigation conducted by [51], which examined the impact of teachers on students’ WTC, both studies acknowledge the influential role of educators in shaping students’ communication behaviors. While [51] focused on teacher-related factors such as delay in response, error correction, and choice of discussion topics, our study delves into the effects of a specific pedagogical approach – free discussion panels. Both studies recognize the dynamic nature of WTC, which can be influenced by various internal and external factors. However, our study extends this understanding by exploring the impact of a student-centered approach on WTC, providing insights into the potential benefits of interactive and collaborative language learning environments.

In contrast, the mixed-methodological study by [52] centered on online synchronous group discussions among Farsi-speaking ESL students. Although both studies recognize the dynamic nature of WTC, our investigation differs in terms of methodology and focus. While [52] explores online discussions and their impact on WTC levels, our study concentrates on the effects of face-to-face free discussion panels in language classes. The differences in context and methodology contribute to a nuanced understanding of how various instructional approaches may influence WTC in language learners.

The case study by [54] on student interactions during mindful tasks in an English reading class provides a point of comparison for the mindfulness component of our study. Both studies recognize the potential link between mindfulness and language learning [54]. observed minor differences in interactions between mindful and less mindful groups during critical reading tasks. In our study, the incorporation of mindfulness principles within free discussion panels contributed to learners’ growth mindfulness, emphasizing the holistic nature of language acquisition.

In contrast, the study by [55] focused on social mindfulness, exploring how perceptions of individuals with varying levels of social mindfulness impact cooperation. While our study acknowledges the importance of collaboration and autonomy within free discussion panels, the focus is on individual and collective mindfulness, extending the understanding of mindfulness in language learning beyond the social realm.

Our study’s emphasis on learner autonomy aligns with the investigation by [56], which developed and validated a scale assessing participants’ perceptions of autonomy, among other variables, in Iranian academic settings. Both studies underscore the interconnected relationships among classroom interaction, learner needs, learner autonomy, pedagogical scaffolding, and learner identity. However, our study specifically explores the impact of integrating free discussion panels on autonomy, providing insights into how learner-centered approaches may foster autonomy in language learners.

Contrastingly, [57] scrutinized ESL learners facing challenges in regular interaction with native speakers and explored the influence on autonomy. While both studies acknowledge the impact of interaction on language learning and autonomy, our study focuses on a specific pedagogical approach – free discussion panels – and its potential to cultivate learner autonomy within a classroom setting.

Our study aligns with the theoretical underpinnings of integrating panel discussions into language education. Rooted in communication theory, SCT, and learner-centered pedagogy, our findings support the notion that panel discussions provide a platform for authentic conversations, aligning with Bakhtin’s dialogism [19]. The active participation, negotiation of meaning, and exposure to diverse linguistic expressions observed in our study reflect the principles of dialogic language development.

Furthermore, the sociocultural nature of learning, emphasized by Vygotsky’s SCT [20], resonates with our study’s practice of panel discussions as a means of fostering collaborative discourse. The shared construction of knowledge through group interaction mirrors the idea that language learning is deeply connected to meaningful social exchanges. Additionally, the learner-centered pedagogy embedded in our study, where teachers serve as facilitators promoting student engagement and critical thinking, aligns with educational theorists like Dewey [21] and Knowles [22].

The concept of turn-taking, integral to conversation analysis [23], is substantiated in our findings. The emphasis on conversational democracy, equal opportunities for participants, and the development of effective communication skills through turn-taking aligns with our study’s exploration of conversational dynamics, active listening, and thoughtful responses—a crucial aspect of language proficiency.

Finally, our findings support the integration of panel discussions as a strategy to foster learner autonomy [24]. Opportunities for peer collaboration, peer review, and individual perspectives within panel discussions empower learners to take ownership of their language learning journey. This resonates with the broader notion of autonomy in language learning, involving active participation in decision-making processes and self-regulated learning.

Our study’s findings concerning WTC are in line with the theoretical foundations laid by Burgoon [25] and subsequent scholars [26–30]. Recognizing the significance of the desire to communicate, our study provides practical insights into the active engagement in communication using the target language, addressing the fundamental objective of language teaching. The contextual exploration of WTC variability under different influences, as highlighted by [28], further enriches the understanding of this dynamic trait.

The incorporation of mindfulness principles within panel discussions aligns with the theoretical background drawing from [31–33]. Our study acknowledges mindfulness as both a process and an outcome, emphasizing intentional attention in the present moment without judgment. The structured program within our study, involving intention, attention, and attitude, resonates with the cultivation of skills like sustained focus, acceptance, discernment, and compassion.

The notion that mindfulness enables a more flexible creation of autobiographical meaning, enhancing individuals’ ability to positively reflect on experiences, is supported by our findings. The observed benefits of mindfulness practice, including well-being, strengthened interpersonal connections, stress alleviation, and prevention of burnout, correspond with the outcomes expected from incorporating mindfulness in language education.

Our study’s exploration of autonomy in language learning is consistent with the theoretical background grounded in the SDT [39–41]. The definition of autonomy as behavior aligned with personal beliefs, genuine interests, and values aligns with our study’s focus on fostering autonomy within language learners.

The significant connection between autonomy and motivation, especially in online learning environments, as affirmed by numerous studies [42–46], is supported by our findings. The acknowledgment of the digital learning landscape as a potential enhancer of learners’ autonomy, while highlighting its affordances, also notes the caution regarding the potential risks, aligning with the broader discourse on technology in education.

This study holds significant implications for language teachers, offering insights that can inform and enhance their instructional practices. The integration of panel discussions into language education, as supported by our findings, underscores the importance of creating authentic conversational opportunities. Language teachers can adopt facilitative roles, encouraging active participation, negotiation of meaning, and exposure to diverse linguistic expressions. Embracing learner-centered pedagogy and turn-taking practices can contribute to the development of effective communication skills. Moreover, the emphasis on fostering learner autonomy through peer collaboration, peer review, and individual perspectives suggests that teachers play a crucial role as facilitators in empowering learners to take ownership of their language learning journey. Language teachers could benefit from the results of this study by incorporating free discussion panels into their teaching practices to promote authentic communication and enhance language learning outcomes. By adopting a learner-centered approach and creating opportunities for open dialogue and collaborative interaction, teachers can cultivate an engaging and supportive learning environment that fosters students’ willingness to communicate, growth mindfulness, and autonomy.

Policy-makers in the realm of language education can draw important implications from this study to shape comprehensive language learning policies. Recognizing the positive impact of panel discussions on language development, policy-makers may consider promoting learner-centered approaches within educational frameworks. Policies that encourage the integration of sociocultural theories, mindfulness practices, and autonomy-building strategies can contribute to creating dynamic and effective language learning environments. The study advocates for policies that support the professional development of language teachers, fostering the skills necessary to implement innovative pedagogical methods aligned with the findings. Policymakers could use the results to shape comprehensive language learning policies that promote learner-centered approaches within educational frameworks. Policies that encourage the integration of sociocultural theories, mindfulness practices, and autonomy-building strategies can contribute to creating dynamic and effective language learning environments that cater to the diverse needs of language learners. Additionally, policymakers could advocate for the professional development of language teachers, fostering the skills necessary to implement innovative pedagogical methods aligned with the findings of this study.

Syllabus-designers play a pivotal role in shaping the curriculum that guides language learning. Based on the study’s findings, syllabus-designers can incorporate panel discussions as a core element in language education programs. This involves delineating specific modules that emphasize authentic conversation, negotiation of meaning, and exposure to diverse linguistic expressions. Designing syllabi that align with the principles of learner-centered pedagogy and conversation analysis can contribute to creating engaging and effective language courses. Additionally, integrating mindfulness practices and strategies to foster learner autonomy can be incorporated into syllabi to enhance the overall language learning experience. Syllabus designers might use the findings to integrate discussion panels as a core element in language education programs, emphasizing authentic conversation, negotiation of meaning, and exposure to diverse linguistic expressions. By designing syllabi that align with the principles of learner-centered pedagogy and conversation analysis, syllabus designers can contribute to creating engaging and effective language courses that prioritize the sociocultural and affective dimensions of language learning.

Materials developers can benefit from the study’s implications by aligning their resources with the identified effective language teaching strategies. Developing instructional materials that facilitate panel discussions, encourage turn-taking, and provide opportunities for collaborative discourse can enhance the communicative competence of learners. Mindfulness practices and autonomy-building resources can be integrated into materials to address the holistic development of language learners. The study advocates for materials that not only focus on linguistic aspects but also prioritize the sociocultural and affective dimensions of language learning. By aligning materials with these implications, developers contribute to creating a more engaging, inclusive, and effective language learning experience. Materials developers can leverage the findings of this study to create instructional materials that facilitate free discussion panels and enhance language learning experiences. By designing materials that encourage turn-taking, collaborative discourse, and authentic communication, developers can support the development of effective communication skills among language learners. Integrating mindfulness practices and autonomy-building resources into materials can address the holistic needs of language learners and promote their cognitive and affective development. Furthermore, materials developers can prioritize the sociocultural and affective dimensions of language learning by designing resources that not only focus on linguistic aspects but also foster learners’ growth mindfulness and autonomy. By aligning materials with the findings of this study, developers can contribute to creating a more engaging, inclusive, and effective language learning experience for learners of diverse backgrounds and proficiency levels.

## Conclusion

In conclusion, this study delved into the impact of integrating panel discussions into language education, exploring its theoretical underpinnings in communication theory, sociocultural theory, and learner-centered pedagogy. Our findings highlight the positive influence of panel discussions on language development, emphasizing active participation, negotiation of meaning, and exposure to diverse linguistic expressions. The study underscores the importance of learner-centered approaches, drawing on sociocultural theories and conversation analysis to promote effective communication skills. Furthermore, the cultivation of learner autonomy through peer collaboration and mindfulness practices emerges as a key aspect of panel discussions.

The study’s design, which employed qualitative methods and purposive sampling, provided a nuanced understanding of the experiences of learners in different conditions. By comparing an experimental group exposed to discussion panels with a control group receiving traditional teacher-fronted lessons, the research sheds light on the potential of innovative pedagogical methods.

In essence, this study contributes to the ongoing discourse on effective language education by advocating for dynamic, engaging, and holistic approaches. The integration of panel discussions, rooted in theoretical frameworks, has the potential to transform language learning environments, fostering not only linguistic proficiency but also sociocultural awareness and learner autonomy. As educators and stakeholders navigate the evolving landscape of language education, these findings provide valuable insights for shaping future pedagogical practices and policies that cater to the diverse needs of language learners.

While this study contributes valuable insights into the influence of discussion panels on language education, it is imperative to acknowledge several limitations that may impact the generalizability and robustness of the findings. Firstly, the qualitative nature of the study and its specific focus on learners in Hebei, China, may restrict the applicability of the results to other educational contexts. Furthermore, the utilization of purposive sampling raises concerns regarding potential biases, as participants were selected based on known language proficiency levels. Additionally, the study’s concentration on intermediate and upper-intermediate levels might not fully capture the nuances of how discussion panels affect learners at varying proficiency levels. The reliance on self-reporting for measures such as WTC and growth mindfulness introduces the possibility of social desirability bias, potentially skewing participants’ responses towards what they perceive as favorable. Lastly, the relatively short duration of the study and the absence of a longitudinal component limit insights into the long-term effects of panel discussions on language development and attitudes. These limitations should be transparently addressed to ensure a comprehensive understanding of the study’s scope and implications.

To address these limitations and further enhance the depth of knowledge regarding the impact of discussion panels on language education, future research endeavors could embark on exploring diverse learner populations encompassing various age groups, educational backgrounds, and language learning experiences. By expanding the participant pool to include individuals with different proficiency levels, from beginner to advanced, researchers can gain a more nuanced understanding of how discussion panels influence learners across the language proficiency spectrum. Additionally, longitudinal studies spanning an extended duration could offer invaluable insights into the sustainability and evolution of the observed effects over time, shedding light on the enduring benefits or potential limitations of integrating discussion panels into language education curricula. Furthermore, conducting comparative studies across diverse cultural and linguistic contexts would enrich the breadth of understanding, allowing for a more holistic assessment of the generalizability and applicability of the findings. Such cross-cultural investigations could elucidate how cultural nuances and language-specific factors may interact with the implementation of discussion panels, informing tailored approaches to language pedagogy that resonate with diverse learner populations worldwide. Exploring how the effects of discussion panels evolve or change over an extended period could provide valuable insights into their long-term impact on language learning outcomes. Longitudinal studies would allow researchers to track participants’ progress and development over time, revealing whether the benefits of discussion panels are sustained or diminish over time. Understanding the trajectory of language learning outcomes resulting from engagement in discussion panels could inform the design of more effective and sustainable language education interventions, guiding educators in optimizing instructional practices for long-term proficiency gains.

Moreover, researchers may consider incorporating quantitative measures alongside qualitative methods to triangulate results and provide a more comprehensive perspective. Investigating the potential role of teacher training in effectively implementing discussion panels and its impact on learner outcomes could also be a fruitful avenue for future research.

The suggestions for further research stem from the recognition of several limitations and gaps in the current study, aiming to address these gaps and advance our understanding of the impact of discussion panels on language education. For example, exploring diverse learner populations and proficiency levels would allow for a more comprehensive understanding of how different learners respond to discussion panels, thereby enhancing the generalizability of the findings. Longitudinal studies could provide insights into the sustainability of the observed effects over time, shedding light on the long-term impact of discussion panels on language development and attitudes. Comparative studies across different cultural and linguistic contexts would help elucidate the cultural factors that may influence the effectiveness of discussion panels, informing more culturally sensitive pedagogical practices. Additionally, incorporating quantitative measures alongside qualitative methods would provide a more comprehensive perspective on the impact of discussion panels, allowing for quantitative validation of qualitative findings. Investigating the potential role of teacher training in effectively implementing discussion panels and its impact on learner outcomes could inform professional development initiatives for language educators, ensuring the successful implementation of discussion panels in diverse educational contexts. Overall, these suggestions aim to address existing gaps in the literature and further our understanding of the role of discussion panels in language education practice.

Exploring the use of technology in facilitating virtual discussion panels and its impact on language learning outcomes could address the growing influence of digital tools in education. Finally, examining the transferability of findings to different language skills and assessing the potential impact on other cognitive and affective domains could contribute to a holistic understanding of the benefits of innovative pedagogical practices.

## Data Availability

The dataset of the present study is available upon request from the corresponding author.
